# State of the Art on Approved Cystic Fibrosis Transmembrane Conductance Regulator (*CFTR*) Modulators and Triple-Combination Therapy

**DOI:** 10.3390/ph14090928

**Published:** 2021-09-15

**Authors:** Aniello Meoli, Valentina Fainardi, Michela Deolmi, Giulia Chiopris, Francesca Marinelli, Caterina Caminiti, Susanna Esposito, Giovanna Pisi

**Affiliations:** 1 Paediatric Clinic, Department of Medicine and Surgery, University Hospital of Parma, Via Gramsci 14, 43126 Parma, Italy; nello.meoli@gmail.com (A.M.); valentina.fainardi@unipr.it (V.F.); michela.deolmi@studenti.unipr.it (M.D.); giulia.chiopris@gmail.com (G.C.); francesca.marinelli@studenti.unipr.it (F.M.); gpisi@ao.pr.it (G.P.); 2 Research and Innovation Unit, University Hospital of Parma, Via Gramsci 14, 43126 Parma, Italy; ccaminiti@ao.pr.it

**Keywords:** *CFTR*, *CFTR* modulator, cystic fibrosis, F508del, ivacaftor

## Abstract

Cystic fibrosis (CF) is the most common life-limiting inherited disease in Caucasian populations, affecting approximately 80,000 people worldwide. CF is a complex multi-organ monogenic autosomal recessive disorder caused by a mutation in cystic fibrosis transmembrane conductance regulator (*CFTR*) gene. Since the discovery of the *CFTR* gene in 1989, more than 2000 mutations have been identified so far and about 240 can cause CF. Until recently, the treatment for CF was aimed to prevent and manage the manifestations of *CFTR* dysfunction, primarily recurrent pulmonary infections and pancreatic exocrine failure. Over the past few decades, the therapeutic approach to CF has been revolutionized by the development of a new class of small molecules called *CFTR* modulators that target specific defects caused by mutations in the *CFTR* gene. *CFTR* modulators have been shown to change profoundly the clinical course of the CF, leading to meaningful improvements in the lives of a large proportion of people of CF heterozygous for F508del, especially if started in young children. Further studies are needed to extend the use of triple *CFTR* modulation therapy also for young children in order to prevent the irreversible effects of the disease and for patients with very rare mutations with a personalized approach to treatment.

## 1. Introduction

**Figure 1:**
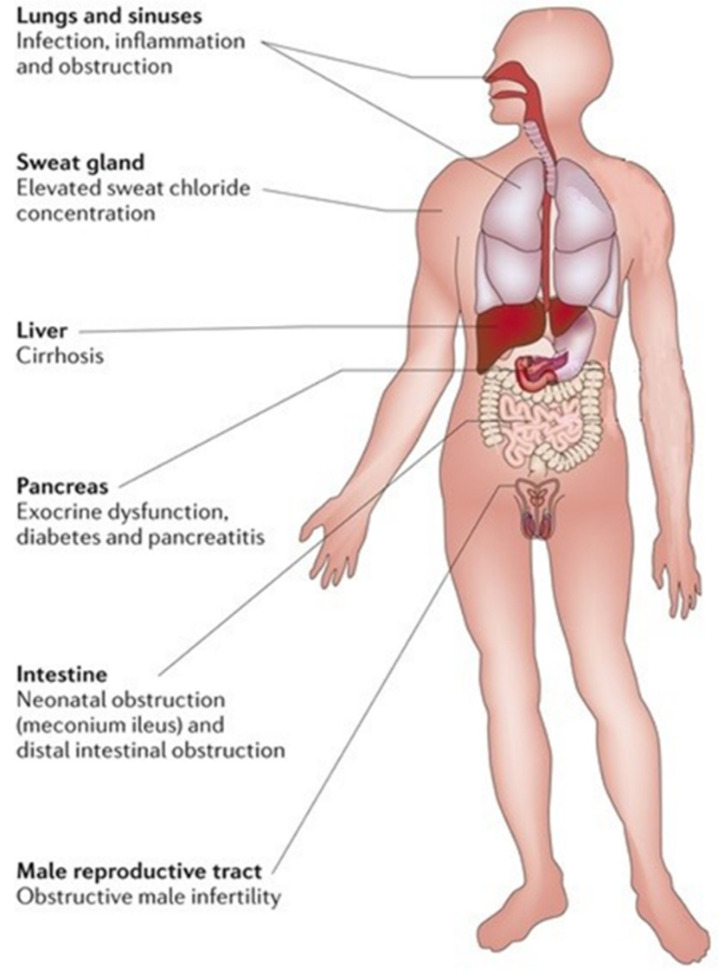
Clinical manifestations of cystic fibrosis.

Cystic fibrosis (CF) is the most common life-limiting inherited disease in Caucasian populations, affecting approximately 80,000 people worldwide [1,2]. CF is a complex multi-organ monogenic autosomal recessive disorder caused by a mutation in cystic fibrosis transmembrane conductance regulator (*CFTR*) gene on the long arm of chromosome 7 [2]. This gene encodes for an anion channel member of the ATP-binding cassette (ABC) proteins regulated by cyclic adenosine monophosphate (cAMP) and protein kinase A (PKA)-dependent phosphorylation responsible for chloride and bicarbonate passive transport through the apical membrane of epithelial cells. The *CFTR* protein is a is 1480 amino acid glycoprotein located on secretory epithelia of airways, pancreas, sweat glands, and the gastrointestinal and reproductive tracts, also involved in the modulation of other adjacent ion channels and therefore crucial for the homeostasis of these organs due to its ability to regulate epithelial surface hydration and luminal pH. A defective *CFTR* function causes a reduced anion secretion through the lumen of airways and digestive system with consequent impaired hydration of their secretions. In addition, in airways, sodium absorption, of which the *CFTR* protein is a down-regulator, contributes to the surface dehydration and impaired mucociliary clearance observed in CF patients. By contrast, *CFTR* mutation in sweat glands causes a reduction in chloride and sodium absorption through the cytoplasm with higher sodium chloride levels in sweat. Hence, impaired function of the *CFTR* gene causes a wide range of symptoms, including thickened mucous with formation of mucus plugs, severe lung infections, pancreatic insufficiency, liver damage, increased sweat chloride concentration, malabsorption with malnutrition, and infertility [2]. Airways involvement with recurrent pulmonary exacerbations, especially by *Pseudomonas aeruginosa* infection that led to progressive lung damage, represents the most frequent cause of early morbidity, death, and need for lung transplantation in CF patients [2]. The reduced bicarbonate secretion in pancreatic ducts of CF patients leads to an increased mucus viscosity and decreased solubility of secreted digestive enzymes with premature activation of them and consequent pancreas destruction [2]. The loss of pancreatic exocrine function leads to the need to take enzyme supplements; over time, the pancreatic damage also affects the endocrine function of these patients that may develop insulin-dependent diabetes mellitus. In addition, the reduced secretion of bicarbonate is also present in the intestinal lumen with increased acidity and consequent epithelial damage and dehydration, which can lead to intestinal obstruction [2]. In neonates, intestinal obstruction may cause meconium ileus, while in adults, it may result in recurrent episodes of distal intestinal obstructive syndrome (DIOS) [2]. Therefore, intestinal dysbiosis may cause chronic diarrhea and weight loss. Figure 1 summarizes the clinical manifestations in patients with CF.

**Figure 2:**
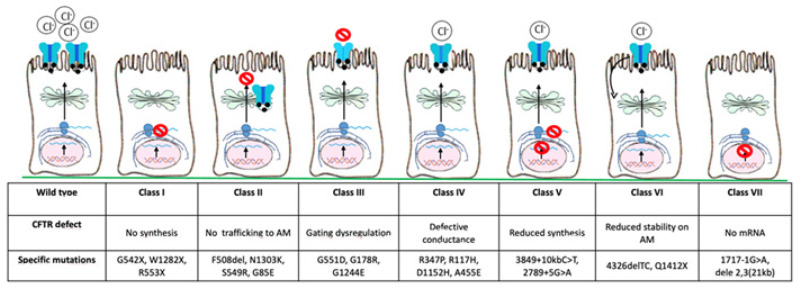
*CFTR* gene mutations are classified into seven main classes based on the defect of *CFTR* gene. AM, apical membrane.

Since the discovery of the *CFTR* gene in 1989, more than 2000 mutations have been identified so far and about 240 can cause CF [3]. Based on the pathophysiological effects of individual variants, they have been classified into seven classes (Figure 2): class I, severely reduced protein synthesis with absent *CFTR* gene expression on the apical membrane; class II, impaired protein processing, folding, or trafficking, and premature degradation with severely reduced *CFTR* gene expression, such as F508del, the most common mutation, especially in North European countries (80–90%); class III, *CFTR* gene can reach the apical membrane, but the channel activation is abnormal (gating mutations); class IV, normal *CFTR* gene expression with reduced conductivity; class V, lower *CFTR* gene expression due to reduced protein synthesis; class VI, lower *CFTR* gene expression due to increased turnover; class VII, no mRNA synthesis and consequently no *CFTR* gene expression [3,4,5]. There is a correlation between *CFTR* gene function and clinical phenotype, with classes I–III and VII resulting in more severe disease, while classes IV–VI may have milder phenotype [6].

Until recently, the treatment for CF was aimed to prevent and manage the manifestations of *CFTR* gene dysfunction, primarily recurrent pulmonary infections and pancreatic exocrine failure [4]. The major and life-threatening consequences of CF are pulmonary infections and progressive lung function decline. Mucociliary clearance is improved by airway clearance techniques and inhaled mucolytics such as dornase alpha (rhDNase) and hypertonic saline, while chronic pulmonary infections are treated with inhaled, oral, and parenteral antibiotics [4]. With the onset of respiratory failure, lung transplant may represent the last therapeutic option. Pancreatic insufficiency is managed by pancreatic enzyme replacement therapy (PERT) and a highly caloric and salty diet, with the supplementation of fat-soluble vitamins; insulin may be needed in patients who develop diabetes mellitus or glucose intolerance [1]. With improved management, life expectancy for people with CF has progressively increased and the median predicted life expectancy is now nearly 50 years [7].

Over the past few decades, the therapeutic approach to CF has been revolutionized by the development of a new class of small molecules called “*CFTR* modulators” that target specific defects caused by mutations in the *CFTR* gene. According to their mechanism of action, *CFTR* modulators can be classified as potentiators (i.e., drugs improving the ions flux through *CFTR* channel already expressed on apical membrane maintaining it in an open status, effective in patients with gating class III and IV mutations), correctors (i.e., drugs facilitating *CFTR* protein folding, processing, and trafficking to the cell surface, effective in patients with class II mutations), amplifiers (i.e., drugs increasing the expression of protein substrate [*CFTR* mRNA] and consequently, synthesis of *CFTR* protein), read-through agents (i.e., drugs promoting the ribosomal over-reading of premature termination codons [PTCs, Class I mutations] enabling the incorporation of an amino acid, thereby increasing the production of *CFTR* proteins), and stabilizers (i.e., drugs rescuing the protein stability at the plasma membrane) [8,9].

This paper aims to critically analyze the role of *CFTR* modulators in CF treatment. We review PubMed literature published from April 2016 to May 2021, using the keywords “cystic fibrosis”, “*CFTR* modulators”, and “combination therapy”. We screened 122 articles and abstracts and then we focused on reviews, meta-analysis, and original articles including randomized controlled trials (RCT) and preclinical studies of potentiators and correctors.

## 2. *CTFR* Potentiators

### 2.1. Ivacaftor (Formerly VX-770; KALYDECO^®^)

**Figure 3:**
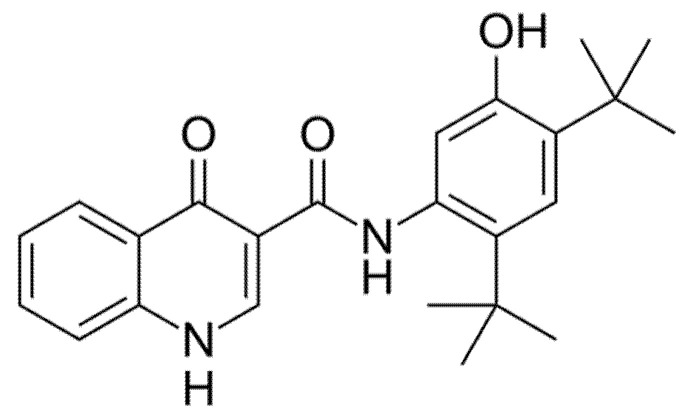
Chemical structure of Ivacaftor.

Ivacaftor (formerly VX-770, Figure 3) was the first *CFTR* modulator approved by the Food and Drug Administration (FDA) in 2012 with the name of Kalydeco^®^ for the treatment of CF patients heterozygous for the G551D, the most common variant of class III. The mechanism of action of Ivacaftor consists of increasing the time of *CFTR* channel in open status through the *CFTR* channel potentiation in a phosphorylation-dependent and ATP-independent manner [10]. Ivacaftor has now been approved for patients older than 4 months with certain class III (G551D, G1244E, G1349D, G178R, G551S, S1251N, S1255P, S549N, S549R) or class IV mutations (R117H), which account for 8% of all patients with CF [11,12].

The first double-blind randomized controlled trial (RCT; STRIVE, NCT00909532) was conducted in 2011 by Ramsey et al. to assess the efficacy of Ivacaftor in 161 patients older than 12 years with at least one G551D mutation for 48 weeks. At week 24, the authors found an improvement of percent predicted Forced Expiratory Volume at 1st second (ppFEV1) by 10.6% in the Ivacaftor group. In addition, they also demonstrated a significant reduction in pulmonary exacerbations (−55%), an improvement in patient-reported respiratory symptoms measured by the Cystic Fibrosis Questionnaire-Revised (CFQ-R), a weight gain on average of 2.7 kg per patient, and a reduction in the concentration of sweat chloride (−48.1 mmol per liter). The incidence of adverse events (AE) was similar with Ivacaftor and placebo, with a lower proportion of serious adverse events (SAE) in Ivacaftor group than placebo group (24% vs. 42%) [13]. Two years later, Ivacaftor was tested among 52 CF children aged 6–11 years with at least one G551D mutation (ENVISION trial, NCT00909727), showing the same promising results observed in adult patients: significant increase in ppFEV1 (+12.6% in the treatment group), weight gain, and reduction in sweat chloride concentration. Compared to placebo, patients treated with Ivacaftor showed no higher risk of AEs. Unlike the STRIVE study, the ENVISION trial did not show a significant improvement in either CFQ-R score or rate of pulmonary exacerbations [11,14].

Thereafter, De Boeck et al. explored the efficacy of Ivacaftor in patients older than 6 years with nine different non-G551D gating mutation in the KONNECTION study (NCT1614470) [15]. After eight weeks of Ivacaftor, authors reported a significant increase in ppFEV1 in the Ivacaftor group compared to the placebo group (+10.7%). A significant improvement was also observed in body mass index (BMI) as well as in concentrations of sweat chloride and CFQ-R scores. These results were maintained through 24 weeks and Ivacaftor was generally well tolerated. This trial suggested that CF patients with non-G551D gating mutations could also benefit of treatment with Ivacaftor [15].

The KONDUCT trial (NCT01614457) assessed the safety and efficacy of Ivacaftor in 69 CF patients aged 6 years or older with at least one copy of the R117H-*CFTR* mutation, which is present in 3% of all CF patients and produces defects in both gating and conductance (class III and IV mutations, respectively) [16]. After 24 weeks of treatment, significant improvements from baseline were found of either CFQ-R score and sweat chloride levels in Ivacaftor group. In contrast, no significant difference from baseline was seen in either ppFEV1 or BMI. However, subgroup analysis based on age showed significant improvement in ppFEV1 in the Ivacaftor group only for patients older than 18 years. These contrasting results between adults and children may be due to the intrinsic nature of R117H-*CFTR* mutation, characterized by a lower degree of severity in children and a delayed onset of significant disease involvement, typically in adulthood [16].

The KIWI trial (NCT01705145), a phase 3 open label study, explored efficacy and safety of Ivacaftor for 24 weeks in 33 children aged 2–5 years with a gating mutation [17]. A significant improvement in sweat chloride concentration and nutritional status was observed, similar to that seen in adult population. No statistical relevant data on pulmonary effect were available due to small age of patients. At baseline, fecal elastase-1 was insufficient in 96.3% of patients but increased above the cut-off for pancreatic insufficiency in 25% of them, suggesting that when started early in life, Ivacaftor could potentially restore exocrine pancreatic function. A higher incidence of liver function test (LFT) elevation was reported in this pediatric study than in adult ones [17].

The KLIMB study (NCT01946412) included 28 of the 33 children enrolled in the KIWI trial and followed them up for a total of 84 weeks [18]. This long-term extension trial proved that Ivacaftor was generally well tolerated in CF children 2 to 5 years old, although even in this study, 30% of children experienced an LFT elevation > 3 × Upper Limit of Normal (ULN) at least on one occasion. Only one child experienced value of alanine aminotransferase > 8 × ULN and required treatment discontinuation. Consistently with the KIWI study, LFT elevation occurred more often in patients with a previous history of transaminase elevation; therefore, authors recommended LFT monitoring before and during treatment with Ivacaftor in children aged 2–5 years. During the 84 weeks of observation, improvements in sweat chloride concentrations, BMI z-scores, and pancreatic function as recorded in the KIWI trial were generally maintained [18].

Safety and efficacy of Ivacaftor in children aged 12–24 months (26 children with at least one *CTFR* gating mutation) was assessed by the ARRIVAL study (NCT02725567), published in 2019 [19]. Oral Ivacaftor was administrated for 24 weeks with rapid and sustained improvement in sweat chloride concentration. Growth parameters were normal at baseline and at week 24. Furthermore, authors observed a significant improvement in fecal elastase-1 and a decrease from baseline in serum lipase and amylase. Once again, improvements in biomarkers of pancreatic function suggest that Ivacaftor, if started early, may preserve exocrine pancreatic function. The most frequently AEs were cough and transaminase elevation; one child suffered from constipation, possibly related to the modulator. No treatment discontinuation or interruption occurred.

Based on in vitro studies, another extension of Ivacaftor has been recently approved by FDA to include *CFTR* gene mutations with residual function (RF), totalizing 28 eligible CF-causing mutations and increasing the number of patients who may benefit from Ivacaftor treatment [20]. *CFTR* gene with RF can arise from a variety of molecular defects, resulting in sufficient *CFTR* protein quantity and/or function to allow some ion transport; therefore, RF mutations are often associated with pancreatic sufficiency and symptoms onset generally occurs at a later age in people with these mutations than in people homozygous for the F508del mutation [21,22]. However, the rate of disease progression in adulthood is similar regardless of *CFTR* genotype and people with CF who have RF mutations may develop severe lung disease.

In 2020, Salvatore et al. published the results of a compassionate use program of Ivacaftor in 26 Italian CF patients with advanced disease carrying at least one RF mutation. This study showed that treatment with Ivacaftor is safe and results in a clinically significant reduction in antibiotic use and improvement in both lung function and walking distance, even in CF patients with severe lung disease [23].

Two of the most common RF, 3849 + 10 kb C→T and D1152H, are associated with Ashkenazi Jewish ancestry and are more common in Israel. Recently, Kerem et al. evaluated the effect of two 8-week treatments period of Ivacaftor in 38 people with CF aged > 6 years with 3849 + 10 kb C→T or D1152H RF mutations [24]. This placebo-controlled crossover study (NCT03068312) confirmed that Ivacaftor treatment resulted in improvements over placebo in lung function measured by Lung Clearance Index (LCI), sweat chloride concentrations, ppFEV1, and CFQ-R respiratory domain score [24].

Effects of Ivacaftor in patients homozygous for the most common mutation (F508del *CTFR*) were explored in the DISCOVER trial (NCT00953706). After 16 weeks of treatment, no significant change in FEV1, BMI, or CFQ-R score was observed; a small reduction in sweat chloride concentration was reported but not maintained through the next 96 weeks of the study.

In conclusion, Ivacaftor has been shown to affect the life expectancy of CF patients with marked benefits maintained over time, slowing down the respiratory decline, reducing the number of pulmonary exacerbations, and restoring sweat chlorine to normal values. However, Ivacaftor alone is effective only in a small number of CF patients (about 5–8%). No therapeutic effect was observed for class II variants such as F508del, suggesting that a *CFTR* potentiator alone cannot be considered as a best therapeutic option for homozygous F508del-*CFTR* patients. A combination with a corrector is required to facilitate trafficking of the misfolded and prematurely degraded protein to the cell membrane, where a potentiator can rectify the activity defect [25].

### 2.2. D9-Ivacaftor (Formerly VX-561 or CTP-656)

During the 39th European Cystic Fibrosis Conference (June 2016), Vertex Pharmaceuticals submitted results from a phase 1 double-blind placebo-controlled study conducted on a deuterated version of Ivacaftor called VX-561 (formerly CTP-656, D9-Ivacaftor) [26,27]. VX-561 were administered once daily for 7 days and compared to 7 days of Ivacaftor in healthy volunteers. Pharmacokinetic profile of VX-561 was superior concerning half-life, clearance rate, and plasma concentration at 24 h [26]. These promising data allowed progression to a phase 2 clinical trial (NCT03911713) in subjects older than 18 years in order to assess safety and efficacy in patients with *CTFR* gating mutation. The study was completed in August 2020, but results are not yet published [27].

### 2.3. Icenticaftor (Formerly QBW-251)

Icenticaftor is a new *CTFR* potentiator whose safety and efficacy were demonstrated by Kazani et al. in their first-in-human RCT of this molecule (NCT02190604) that included both healthy subjects and CF patients with ≥1 pre-specified *CFTR* Class III or IV mutation, or homozygous for F508del mutation [28]. Over 14 days of intervention period, Icenticaftor was well tolerated among enrolled individuals with no unexpected events or discontinuations in the CF groups; the most frequent AEs in CF patients were nausea (12.2%), headache (10.2%), and fatigue (6.1%). Regarding efficacy, Kazani et al. registered significant improvements in ppFEV1 (+6.46%), LCI2.5 (−1.13 points), and sweat chloride (−8.36 mmol/L) in patients with class III and IV mutations treated with Icenticaftor, while no significant efficacy was observed in patients homozygous for a single F508del. Authors concluded that, similarly to Ivacaftor, monotherapy with Icenticaftor is not clinically sufficient in this subgroup of patients but, based on their results, this molecule might be useful when used in a combination therapeutic with *CFTR* correctors [28].

### 2.4. ABBV-3067 (Formerly GLPG3067)

ABBV-3067 is a new molecule that acts as a *CTFR* potentiator. An ongoing phase 2 clinical trial (NCT03969888) is testing its safety and efficacy both alone and in combination with ABBV-2222 (see below) in adult patients homozygous for the F508del mutation; the estimated completion date of the study is fixed in 2022 [29].

### 2.5. ABBV-974 (Formerly GLPG1837)

ABBV-974 (formerly GLPG1837) is a new generation *CTFR* potentiator, currently in phase 2 clinical trials. SAPHIRA 1 (NCT02707562) is a phase 2 open-label study enrolling subjects with at least one copy of the G551D mutation; SAPHIRA 2 (NCT02690519) is an open-label study testing patients with at least one copy of the S1252N class III mutation [30]. Treatment with GLPG1837 was found to be safe and well tolerated in both trials and a statistically significant and dose-dependent decrease in sweat chloride concentration was registered in all groups in SAPHIRA 1 study [30].

## 3. *CTFR* Correctors

### 3.1. Lumacaftor (Formerly VX-809)

**Figure 4:**
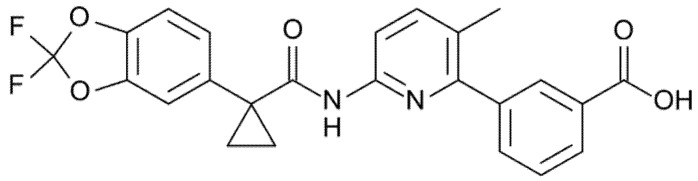
Chemical structure of Lumacaftor.

Lumacaftor (formerly VX-809, Figure 4) was the first approved *CFTR* corrector active on F508del-*CFTR* protein. Its mechanism of action is yet not completely clear, but several studies indicate that it may repair aberrant assembly of the full-length protein, which is mediated through the NBD1:ICL4 interface, and consequently improves processing, trafficking, and stability of the full-length protein. Therefore, Lumacaftor increases the amount of mature *CFTR* at the cell surface [31].

Van Goor et al. studied Lumacaftor’s properties in vitro; human bronchial epithelial (HBE) cells from CF lungs incubated for 48 h with Lumacaftor showed a significant increase in *CTFR* maturation (eightfold) and in transepithelial chloride transport (fourfold). The same study investigated the combination of Lumacaftor with Ivacaftor: the addition of Ivacaftor to cells homozygous for F508del *CTFR* further increased the chloride transport, reaching the equivalent to 25% of non-CF cells [32].

The safety, tolerability, and pharmacodynamics of Lumacaftor as monotherapy was evaluated in adult patients with CF, homozygous for the F508del-*CFTR*, in a 4-week, double-blind RCT (NCT00865904) [33]. Authors reported reductions in sweat chloride concentration but not statistically significant changes in lung function or patient-reported outcomes. AEs were similar in Lumacaftor and placebo groups. Respiratory events were the most frequently reported and caused discontinuation by one subject in each treatment arm [33].

The mutation F508del produces, as already mentioned, a processing and trafficking defect, resulting in premature degradation, as well as defects in gating and stability for *CFTR* that localizes to the cell surface [34]. These characteristics provide an explanation as to why Lumacaftor monotherapy was not effective in patients homozygous for this mutation. Moreover, these characteristics indicate that, to better correct the F508del defect, a *CFTR* potentiator (e.g., Lumacaftor) and corrector (e.g., Ivacaftor) combination should be used.

### 3.2. Tezacaftor (Formerly VX-661)

**Figure 5:**
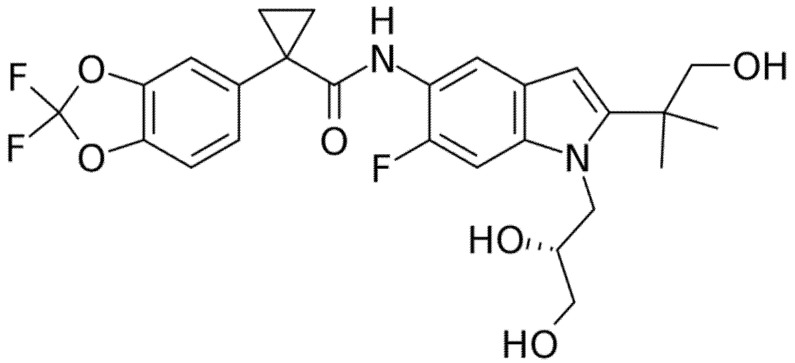
Chemical structure of Tezacaftor.

Tezacaftor (formerly VX-661, Figure 5) is a broad-acting first-generation *CFTR* corrector designed on the basis of the chemical structure of Lumacaftor yet with improved pharmacokinetics and fewer side effects [31,35]. This small molecule, similarly to Lumacaftor, binds the F508del-*CFTR* protein and repairs the aberrant ICL:NBD1 interface, facilitating the intracellular processing and trafficking of normal *CFTR* gene and multiple mutant *CFTR* forms (including F508del), thereby increasing the amount of *CFTR* protein at the cell surface and resulting in an enhanced chloride transport [31,35].

In an in vitro study in F/F HBE cells, Tezacaftor in monotherapy and in combination with Ivacaftor was able to improve chloride transport and enhance fluid transport and ciliary beat frequency [36].

The safety and efficacy of Tezacaftor were evaluated by Donaldson et al. in a randomized, placebo-controlled, double-blind, multicenter, phase 2 study (NCT01531673) in subjects with F/F and F508del/G551D genotypes [35]. According to study design, F/F patients (at least 18 years of age) received Tezacaftor alone or in combination with Ivacaftor. Tezacaftor in monotherapy led to some improvement in lung function in F508del homozygous patients, slightly higher than Lumacaftor [33]. Regarding safety, this study demonstrated a comparable profile across treatment arms with low rates of discontinuation. The majority of AEs among all groups were mild to moderate in nature and the most common were infective pulmonary exacerbations, cough, increased sputum, headache, fatigue, nausea, and diarrhea. The incidence of SAEs was higher in the placebo group, consistently with the higher number of pulmonary exacerbations.

### 3.3. Galicaftor (Formerly GLPG2222 or ABBV-2222)

Galicaftor (formerly GLPG2222) is a novel, potent *CFTR* corrector restoring the processing and trafficking of the mutated *CFTR* gene to the plasma membrane. In HBE cells from F/F homozygous subjects, Galicaftor in combination with a potentiator partially restored *CFTR* function was over 25-fold more potent than Lumacaftor [37]. In 2016, a first human study was conducted to assess safety and pharmacokinetics of ABBV-2222 in healthy adult subjects, reporting promising data [38].

Subsequently, two placebo-controlled phase 2 studies evaluated this molecule in adult F/F patients (FLAMINGO trial, NCT03119649) and in patients heterozygous for F508del *CTFR* carrying a gating class III mutation, who were receiving Ivacaftor (ALBATROSS trial, NCT03045523) [39]. Results showed a decrease in sweat chloride concentration but no significant changes in pulmonary function or respiratory symptoms; relative to the safety, Galicaftor was well tolerated [39]. Further studies are needed in order to assess its contribution in restoring CTFR function.

### 3.4. VX-152, VX-440, VX-445, and VX-659

VX-152, VX-440, VX-445, and VX-659 are additional next generation *CTFR* correctors, used in triple combination with Ivacaftor and Tezacaftor (see Section 4).

### 3.5. FDL 169

FDL 169 is a *CTFR* corrector able to rescue, in F508del HBE cells, *CFTR* expression with similar efficacy as Lumacaftor. FDL 169 is currently in phase 1–2 trial (NCT03093714, NCT02767297) conducted on subjects aged 18 years and older homozygous for the F508del-*CFTR* mutation, whose results have not yet been published [40,41].

## 4. Double Combination *CFTR* Modulator Therapy

### 4.1. Lumacaftor + Ivacaftor (ORKAMBI^®^)

Orkambi^®^ was the first approved combination of a *CFTR* corrector (Lumacaftor) and potentiator (Ivacaftor) by FDA and European Medical Agency (EMA) in 2015. It is indicated in children aged 2 years and older who are homozygous for F508del-*CFTR* mutation [42]. On one hand, Lumacaftor helps in moving the defective protein to its correct site; on the other hand, Ivacaftor increases the conductance of ions and fluid, powering the protein function.

A phase 2 RCT in patients older than 18 years and a longer (24 weeks) phase 3 RCT in patients older than 12 years (TRAFFIC and TRANSPORT studies, NCT01807923 and NCT01807949, respectively) assessing the safety and efficacy of Lumacaftor/Ivacaftor (LUM/IVA) combination showed slight but significant positive effects, notably on lung function (increase in ppFEV1 ~3–4% from baseline) for F508del homozygous patients, together with a significant increase in BMI and a reduced number of exacerbations (30–39% lower than in placebo) as well as hospitalizations [43,44]. The incidence of AEs was generally similar in the LUM/IVA and placebo groups [43,44]. Patients who completed these studies were progressed to 48 weeks in the PROGRESS trial (NCT01931839) and results in the long term were consistent with the TRAFFIC and TRANSPORT trials, confirming the safety and the benefits with a 42% slower rate of ppFEV1 decline, reduction in the incidence of pulmonary exacerbations, and improvement of BMI in LUM/IVA group [45]. The most common AEs in patients treated with LUM/IVA were infective pulmonary exacerbations, cough, headache, dyspnea, chest tightness, hemoptysis, and increase in sputum production. Four percent of patients treated with LUM/IVA discontinued therapy due to AE versus 1.6% in the placebo group. Abnormal LFTs and increased blood pressure were also reported but did not lead to severe AEs [44,45].

The aforementioned phase 2 study included a small cohort of F508del heterozygous people who showed no clinical benefit when taking the LUM/IVA combination [43]. This suggests that combination therapy does not provide benefit in F508del heterozygotes unless the second mutation is responsive to Ivacaftor alone (for example, class III mutations). A phase 3b open-label prospective study (NCT02390219) evaluating the benefit of LUM/IVA for patients with advanced lung disease (ppFEV1 < 40%) observed more frequent respiratory AEs and therefore recommended treatment initiation at a lower dose [46].

Subsequent phase 3 studies of LUM/IVA in children aged 6 to 11 years homozygous for the F508del mutation consistently confirmed a significant improvement in lung function with a decrease in LCI2.5 and increase in FEV1 [47,48]. Sweat chloride concentration also decreased significantly, thus providing a *CFTR* biomarker of modulator efficacy; improvements in nutritional status and health-related quality of life were observed after 24 weeks of treatment. LUM/IVA was well tolerated in this young population. Most common AEs in the LUM/IVA group were productive cough, rhinorrhea, nasal congestion, and abdominal and oropharyngeal pain [47,48].

An additional phase 3, open-label, 24-week study (NCT02797132) assessed the safety, pharmacokinetics, pharmacodynamics, and efficacy of LUM/IVA in children aged 2–5 years, weighing at least 8 kg at enrollment, homozygous for F508del-*CFTR* mutation [49]. Results showed a decrease in the mean sweat chloride concentration, increase in growth parameters, and improvement of biomarkers of pancreatic function (fecal elastase-1 levels increased and serum immunoreactive trypsinogen levels decreased). Most children (98%) had at least one AE, but most were mild to moderate in severity. The most common AEs were cough (63%), vomiting (28%), pyrexia (28%), and rhinorrhea (25%). Four subjects (15% of patients) experienced more serious events: two cases of infective pulmonary exacerbation, one of constipation, and one of viral gastroenteritis. Discontinuation of treatment was observed in three children, due to elevation of LFTs levels. Authors concluded that LUM/IVA were generally safe and well tolerated in children aged 2–5 years with CF for 24 weeks; efficacy results also highlight the potential of early intervention with LUM/IVA to modify the course of disease [49].

Real-life data revealed that clinical response may also vary significantly amongst patients with the same genotype; frequent respiratory AEs and drug intolerance which led to discontinuation of the treatment in some cases were also reported [50]. Notably, interaction between Lumacaftor and Ivacaftor can limit the efficacy of the singular drug. Chronic Ivacaftor exposure reduces Lumacaftor-rescued *CFTR* in F508del-expressing cells while Lumacaftor reduces plasma concentration of Ivacaftor through the induction of cytochrome CYP3A4 activity; such findings might partially explain the modest efficacy observed in co-treatment with LUM/IVA in clinical trials [51,52].

### 4.2. Tezacaftor + Ivacaftor (SYMDEKO^®^/SYMKEVI^®^)

A second combination of a *CFTR* corrector (Tezacaftor) and potentiator (Ivacaftor) was approved in the United States by FDA and in Europe by EMA in 2018 with the names of Symdeko^®^ and Symkevi^®^, respectively [53,54]. At present, in Europe, this combination is indicated in subjects aged 6 years and older, homozygous for the F508del mutation or heterozygous for the F508del mutation with one of the following mutations of the *CFTR* gene: P67L, R117C, L206W, R352Q, A455E, D579G, 711 + 3A→G, S945L, S977F, R1070W, D1152H, 2789 + 5G→A, 3272 26A→G, or 3849 + 10kbC→T [53]. In the United States, its use is granted also in subjects with these 12 additional mutations: E56K, R74W, A1067T, E193K, D110H, R347H, D110E, F1052V, F1074L, K1060T, D170N, E831X [54].

Combination therapy with Tezacaftor/Ivacaftor (TEZ/IVA) was evaluated in the EVOLVE trial, a phase 3, randomized, double-blind, multicenter, placebo-controlled, parallel-group study (NCT02347657) in CF patients 12 years of age or older, homozygous for F508del mutation [55]. Through 24 weeks of treatment, significant improvements in mean absolute change in ppFEV1 from baseline (treatment difference + 4.0%) as well as in annual estimated rate of pulmonary exacerbations (−35%) were observed in the investigational over placebo group. Significant improvements in CFQ-R scores and sweat chloride concentration were also reported in the TEZ/IVA group [55]. No difference was found in BMI between the two therapeutic arms.

Relative to the safety, the incidence of AEs was similar in the two groups, with most of them mild to moderate. Similar results both in terms of efficacy (ppFEV1, sweat chloride concentration) and safety were observed by Donaldson et al. in their randomized, placebo-controlled, double-blind, multicenter, phase 2 study (NCT01531673) conducted in subjects homozygous for F508del and treated with TEZ/IVA for 28 days [36]. Therefore, combination treatment with TEZ/IVA in subjects homozygous for F508del mutation demonstrated comparable therapeutic benefit (in terms of sweat chloride concentration and ppFEV1) to those observed with LUM/IVA in the phase 3 TRAFFIC/TRANSPORT studies [35,55].

Furthermore, the TEZ/IVA combination is characterized by an increased tolerability profile it is because not burdened by the respiratory events (e.g., dyspnea, chest tightness) and the acute lung function decline sometimes registered post LUM/IVA initiation [35,44]. In addition, Tezacaftor is characterized by an ameliorated drug–drug interaction (DDI) profile since it is not a CYP3A4 inducer, thereby interfering with the metabolism of Ivacaftor (substrate of CYP3A4 and CYP3A5) or other drugs (e.g., hormonal contraceptives, Rifampin). On the contrary, Lumacaftor induces (CYP3A, CYP2B6, CYP2C8, CYP2C9, CYP2C19) or inhibits (CYP2C8, CYP2C9) several CYP subtypes [35,44,56,57].

In EXPAND study, the efficacy and safety of Ivacaftor alone or in combination with Tezacaftor were evaluated in CF patients 12 years of age or older heterozygous for F508del mutation and a RF-*CFTR* mutation [58]. In this phase 3, multicenter, double-blind, three-intervention crossover RCT (NCT02392234), subjects were randomized to receive either combination treatment (TEZ/IVA), monotherapy with Ivacaftor, or matching placebo. After 8 weeks of treatment, in the combination treatment group, the improvement in ppFEV1 (+6.8% vs. placebo) was statistically higher than that observed in the Ivacaftor monotherapy group (+4.7% vs. placebo). Moreover, a significant improvement in absolute change in CFQ-R score was observed, both in TEZ/IVA group and Ivacaftor alone group (11.1 and of 9.7 points, respectively) compared to placebo. Absolute change in sweat chloride concentration versus placebo was higher in the combination therapy compared to monotherapy. Relative to the safety, the incidence of AEs was similar across intervention groups with most events (cough, infective pulmonary exacerbations, hemoptysis, and headache) mild or moderate in severity [58].

In following phase 3 clinical trials, co-treatment of TEZ/IVA was demonstrated to reduce sweat chloride concentration and preserve lung function in children aged 6–11 years who are homozygous or heterozygous for the F508del-*CFTR* mutation and a RF [59]. Such findings served as a basis for the extended approval of TEZ/IVA for patients aged ≥ 6 years. Extension clinical trials are ongoing to evaluate the longer-term effects of TEZ/IVA (NCT03537651) and the safety and efficacy in younger children [60].

## 5. New *CFTR* Modulators and Triple Combination Therapy

The evidence of a moderate clinical improvement, notably in respiratory function, obtained with both the dual combinations LUM/IVA and TEZ/IVA in F/F patients and the lack of suitable treatment for minimal function (MF) mutations (that does not produce the protein or produces a protein that is not responsive to Ivacaftor or TEZ/IVA combination), have prompted pharmaceutical companies to develop next-generation correctors, targeting different *CFTR* sites to maximize the effect of triple-combination treatments [61]. The backbone for this pharmacological approach, known as triple therapy, is represented by TEZ/IVA for its more favorable pharmacological properties, including lower CYP3A activation [62].

Four novel correctors—VX-152, VX-440, VX-445, and VX-659—demonstrated a pronounced improvement of *CFTR* activity when co-administered with TEZ/IVA in HBE cells (in F/F genotype). In Ussing chamber studies (based on physiological systems used to measure the transport of ions, drugs, and nutrients across epithelial tissues) conducted on HBE cells obtained from F/F homozygous patients, VX-440 and VX-152 in triple combination with Ivacaftor and Tezacaftor restored chloride transport to ~75 and 65% of normal *CFTR* function, respectively [62,63]. In addition, in the same studies using HBE cells collected from CF patients with one copy of F508del and a MF mutation (F/MF), VX-440 and VX-152 in triple combination with Ivacaftor and Tezacaftor restored chloride transport to ~45% and 40% of normal *CFTR* function, respectively [64]. Interestingly, treatment with Ivacaftor/Lumacaftor resulted in chloride transport of ~25% and 15% of normal in HBE cells collected from CF patients with F/F and F/MF genotype, respectively (data shown at 29th NACFC by Vertex Investigator Meeting).

**Figure 6:**
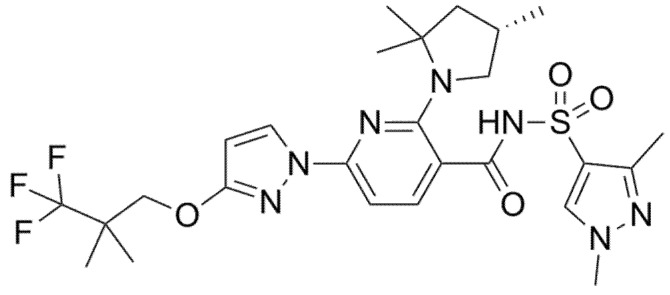
Chemical structure of Elexacaftor.

**Figure 7:**
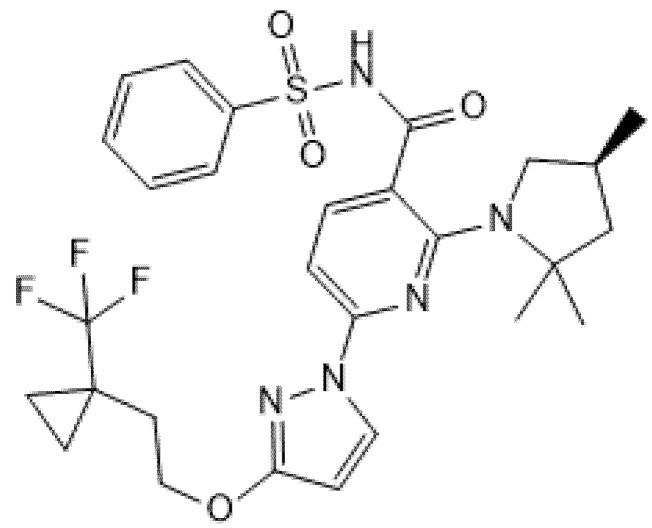
Chemical structure of Bamocaftor.

Two phase 2, double-blind RCTs tested safety and efficacy of triple combination of VX-440 (NCT02951182) or VX-152 (NCT02951195) with Ivacaftor and Tezacaftor in adult F/MF patients and showed a mean increase in ppFEV1 of 12% and 9.7%, respectively [61,62,63,64]. Furthermore, F/F patients who had already been treated with Ivacaftor and Tezacaftor experienced a 7.3% and 9.5% increase in ppFEV1 after addition of VX-152 or VX-440, respectively. VX-445 (Elexacaftor, Figure 6) and VX-659 (Bamocaftor, Figure 7) were selected for subsequent clinical studies because of their pharmacological properties and long-term safety profiles [61,62,63,64].

### 5.1. Elexacaftor (Formerly VX-445)

Elexacaftor (formerly VX-445) was approved in a combination regimen with Tezacaftor and Ivacaftor by the FDA and EMA in October 2019 and in August 2020 under the name of Trikafta^®^ and Kaftrio^®^, respectively [65,66]. This therapy is indicated in subjects aged 12 years and older with F/F and F/MF genotypes (most frequent MF variants: G542X, W1282X, R553X, R1162X, 621 + 1G→T, 1717 − 1G→A 1898 + 1G→A, 3659delC, 394delTT, CFTRdel2,3, N1303K, I507del, G85E, R347P, R560T) [65,66].

The pharmacologic evaluation conducted on HBE cells isolated from CF patients with F/MF and F/F genotypes showed that the combination of Elexacaftor and Tezacaftor, with or without Ivacaftor, increased levels of mature *CFTR* protein and chloride transport (between 60–80% of normal *CFTR*) [67]. These in vitro studies provided the molecular and biologic rationale for investigating the safety and efficacy of Elexacaftor in triple combination with Tezacaftor and Ivacaftor for 4 weeks in a phase 2 double-blind, dose-ranging RCT (NCT03227471) that involved CF patients 18 years of age or older with F/F and F/MF genotypes. In this proof-of-concept clinical trial, the triple combination with Elexacaftor/Tezacaftor/Ivacaftor (ELX/TEZ/IVA) significantly increased the ppFEV1 (primary endpoint) in both population groups up to 13.8% and 11% in F/MF and F/F subjects, respectively [67]. This effect was present at the time of the first measurement after the start of treatment (2 weeks), indicating that the improvements in lung function occurred rapidly, and was maintained for the entire duration of the study. In both groups, there was also a decrease in sweat chloride concentrations up to 39.1 mmol/L and an improvement in the respiratory domain of CFQ-R score up to 25.7 points.

Regarding safety, the ELX/TEZ/IVA combination presented an acceptable and comparable AEs profile among patients with both genotypes; 92% of subjects had an AE, but the majority (97%) were mild or moderate in severity, and the trial drug was interrupted or discontinued because of an AE event in 8% of patients [67]. The most common AEs were cough, increased sputum production, hemoptysis, infective pulmonary exacerbation of CF, and fever while no episodes of acute bronchoconstriction were reported. The incidence of laboratory abnormality was 8% and 3% for elevation of LFT and bilirubin levels, respectively [67].

The remarkable preliminary results of phase 2 studies on Elexacaftor led to the AURORA program that includes the following studies: VX17-445-102 (NCT03525444) and VX17-445-103 (NCT03525548) to assess safety and efficacy in F/MF and F/F populations, respectively, and VX17-445-105 (NCT03525574), an ongoing 96-week open-label extension study to prosecute randomized placebo-controlled (F/MF) or active-controlled (F/F) treatment studies [62]. Heijerman et al., in their phase 3, multicenter, double-blind RCT (VX17-445-103 Trial, NCT03525548), randomized stable CF patients aged 12 years or older with F/F genotype to receive Elexacaftor-based triple combination or Tezacaftor/Ivacaftor (TEZ/IVA) for 4 weeks [68]. Patients who received ELX/TEZ/IVA combination had a significant increase of 10 points in ppFEV1 than patients who received TEZ/IVA therapy. Likewise, in the triple combination group at week 4, sweat chloride concentration decreased by 45.1 mmol/L, resulting in a mean value below the diagnostic threshold for CF, and the CFQ-R respiratory domain improved by 17.4 points, which exceeds the known 4-point improvement corresponding to the minimal clinically significant difference in patients with CF [68]. Although the treatment duration in this trial was only 4 weeks, an increase in BMI was observed in the ELX/TEZ/IVA group compared with participants who received TEZ/IVA therapy. At trial completion, participants were given the option to enroll in a 96-week open-label extension trial (VX17-445-105; NCT03525574). The triple combination regimen was well tolerated, with no discontinuations. Most AEs were mild or moderate; serious AEs occurred in two (4%) participants receiving ELX/TEZ/IVA and in one (2%) receiving TEZ/IVA [68]. The most reported AEs, occurring in more than 10% of participants in either treatment group, were cough, nasopharyngitis, upper respiratory tract infections, and oropharyngeal pain more often in the ELX/TEZ/IVA arm, and pulmonary exacerbation of CF, hemoptysis, and headache in the TEZ/IVA arm. The authors reported an increase in liver enzymes concentrations in only two (4%) participants in ELX/TEZ/IVA and in one (2%) participant in the TEZ/IVA group.

Middleton (VX17-445-102, NCT03525444) conducted a wide phase 3 RCT to confirm the efficacy and safety of ELX/TEZ/IVA in 403 CF patients 12 years of age or older with F/MF genotype for 24 weeks [69]. ELX/TEZ/IVA, compared to placebo, resulted in an increase in ppFEV1 of 13.8 points at 4 weeks and 14.3 points through 24 weeks, an increase in CFQ-R respiratory domain score of 20.2 points, a 63% reduction in rate of pulmonary exacerbations, and a decrease of 41.8 mmol/L of sweat chloride concentration. BMI also improved significantly at week 24, with a mean treatment difference of 1.04 relative to placebo. This study also confirmed that ELX/TEZ/IVA was generally safe and had an acceptable side-effect profile. The percentage of patients with at least one AE was 93.1% in the ELX/TEZ/IVA group and 96.0% in the placebo group. Most patients had AEs that were mild or moderate and discontinuation of the trial occurred in only 2 patients (1%). SAEs occurred in 13.9% in ELX/TEZ/IVA group and in 20.9% in placebo group [69]. AEs occurring in at least 10% of patients in either trial group were consistent with common manifestations and complications of CF: headache, diarrhea, and upper respiratory tract infections were more frequent in triple combination arm, whereas pulmonary exacerbation, cough, sputum increase, hemoptysis, oropharyngeal pain, and fatigue were prevalent in the placebo arm. Elevation of LFTs occurred in 10.9% of the patients in the ELX/TEZ/IVA group and 4.0% in the placebo group; furthermore, rash occurred in 10.9% of the patients in the ELX/TEZ/IVA group and 6.5% in the placebo group. In both trial groups, rash was more common in female patients than in male patients and more common in female patients who used hormonal contraceptives than in those who did not.

CF patients from both pivotal phase 3 studies [68,69] were invited to participate in an ongoing, phase 3, open-label extension (OLE) study to evaluate the long-term safety and efficacy of ELX/TEZ/IVA [70]. The results of this interim analysis were consistent with pivotal phase 3 studies, demonstrating both the safety and sustained efficacy of long-term (24–36 weeks) ELX/TEZ/IVA treatment in patients CF 12 years or older with one or more F508del allele.

Recently, a prospective observational study including 245 patients aged ≥12 years and with ppFEV1 < 40% who received ELX/TEZ/IVA for 9 months was conducted in France [71]. In these patients, an absolute increase in ppFEV1 (+15.1%) and in weight (+4.2 kg) were observed; moreover, the number of patients requiring long-term oxygen, non-invasive ventilation, enteral tube feeding, and lung transplantation significantly decreased, suggesting that ELX/TEZ/IVA is associated with rapid clinical improvement also in patients with advanced respiratory disease, often leading them to suspend the waiting list for lung transplantation [71]. Given the strong clinical improvements observed with ELX/TEZ/IVA treatment in CF patients ≥ 12 years of age, as well as the critical need for more effective *CFTR* modulation in younger patients with at least one F508del allele, recently, Zemanick et al. evaluated the safety and efficacy of ELX/TEZ/IVA in 66 children of 6–11 years of age with F/MF or F/F genotype in a 24-week open-label, multicentric, phase 3 study (VX18-445-106, NCT03691779) [72]. Through week 24, therapy led to improvements consistent with those observed in the controlled phase 3 pivotal studies in older CF patient in AURORA program [68,69] in both genotype cohorts except for sweat chloride concentrations. In this outcome, studied subjects appeared globally more responsive to therapy than older patients but with significative differences between F508del/MF and F508del/F508del genotype: in fact, in the first cohort, the registered sweat chloride decreasing was −55.1 mmol/L while in the second one it was −70.4 mmol/L from baseline. Regarding respiratory outcomes, ELX/TEZ/IVA improved ppFEV1 and LCI2.5 by 9.1 percentage points and −1.72 units in the F508del/MF cohort, respectively, and by 11.2 percentage points and −1.64 units in the F508del/F508del cohort, respectively [72]. Furthermore, ameliorated CFQ-R scores were reported of a magnitude of 6.9 and 7 points in the F508del/MF and F508del/F508del arms, respectively. Sixty-five children (98.5%) had AEs, which for most were mild or moderate and generally consistent with manifestations of CF or common childhood infections; the most common were cough, headache, pyrexia, oropharyngeal pain, and upper respiratory tract infection. Only one child experienced a SAE concurrent with respiratory virus infection. Elevation in LFTs was reported in 10.6% of patients but none of these interrupted or discontinued the study [72]. Rash events occurred in 24.2% of subjects but all of them were mild or moderate in severity and resolved spontaneously; only one patient discontinued the study because of an erythematous rash that developed after the first dose of ELX/TEZ/IVA. Moreover, for twelve of the children (75%), the rash events were assessed as not related to study drug or suggestive of an alternative etiology. To summarize, the study further confirmed the ability of ELX/TEZ/IVA to modulate a single F508del-*CFTR* allele in patients with CF. The long-term safety and efficacy of ELX/TEZ/IVA in CF children who are 6 years of age and older will be assessed in the ongoing 96-week open-label extension study (VX19-445-107, NCT04183790) that enrolled all 64 patients who completed the treatment period of Zemanick et al.’s study [72].

In conclusion, the magnitude of therapeutic responses obtained with ELX/TEZ/IVA triple combination was greater than that achieved with benchmark Ivacaftor in patients with G551D [13,35] or other gating mutations [15], justifying the term of Highly Effective Modulator Treatment (HEMT).

### 5.2. Bamocaftor (Formerly VX-659)

In Ussing chamber studies, Bamocaftor in triple combination with Ivacaftor/Tezacaftor restored chloride transport to ~65% of normal *CFTR* in HBE cells collected from F/MF patients [73]. Similarly to Elexacaftor, after in vitro pharmacologic and biochemical studies on HBE from CF patients with F/MF or F/F genotypes, Bamocaftor in triple combination with TEZ/IVA was tested in a randomized, placebo-controlled, double-blind, multicenter, phase 1 trial (NCT 03029455, VX-16-659-001) conducted to evaluate preliminary pharmacokinetics and initial safety in a small group of healthy volunteers and CF patients. Most AEs were mild or moderate, and none led to interruption of the trial regimen [74].

Subsequently, the safety and efficacy of this molecule were evaluated in a larger, randomized, parallel-track, placebo- or active-controlled, double-blind, multicenter, dose-ranging, phase 2 trial (VX-16-659-101, NCT03224351) enrolling 117 CF patients 18 years of age or older with F/F and F/MF genotype [74]. In this study, Bamocaftor in triple combination therapy showed an acceptable safety profile in both cohorts. The majority of AEs were mild to moderate and the most common were cough, infective pulmonary exacerbation, oropharyngeal pain, headache, and increased sputum production. No episodes of acute bronchoconstriction were reported. Two cases of elevation of LFT were reported in patients who received Bamocaftor/Tezacaftor/Ivacaftor (VX-659/TEZ/IVA) without concurrent elevation in the bilirubin level.

Regarding efficacy, triple combination with VX-659/TEZ/IVA produced at 4 weeks a significant increase in ppFEV1 up to 13.3 and 9.7 percentage points over the comparator in F/MF and F/F cohorts, respectively. Among F/MF and F/F cohorts, respective improvements in absolute change in sweat chloride concentration and CFQ-R scores were up to −51.4 mmol/L and −42.2 mmol/L and up to + 24.6 and + 19.5 points over the comparator in favor of triple combination therapy arm [74].

The robust results of phase 2 studies on Bamocaftor led to the advancement to phase 3 studies (ECLIPSE programs). The ECLIPSE program includes VX17-659-102 (NCT03447249) and VX17-659-103 (NCT03460990) to assess safety and efficacy in F/MF and F/F cohorts, respectively, and VX17-659-105 (NCT03447262) for the ongoing 96-week open-label extension study of the program [62]. A phase 3, randomized, double-blind, controlled study (VX17-659-103 trial) evaluating the efficacy and safety of Bamocaftor-based triple therapy in 116 CF patients with F/F genotype demonstrated their superiority to TEZ/IVA at week 4 both for efficacy and safety [75]. Regarding efficacy, ppFEV1 from baseline at week 4 increased significantly by 9.9 percentage points and sweat chloride concentration decreased significantly by −48.7 mmol/L, under the diagnostic threshold for CF, in the Bamocaftor triple therapy group; in addition, CFQ-R significantly improved by 13.5 points in this cohort. In VX17-659-103 trials, Bamocaftor triple combination therapy demonstrated its safety and tolerability: the percentage of patients with at least one AE was 61.1% in the VX-659/TEZ/IVA group and 54.4% in the TEZ/IVA group [75]. The most frequent AEs were sputum increase, cough, diarrhea in Bamocaftor triple therapy, and infective pulmonary exacerbation and headache in the TEZ/IVA group. Serious AEs were reported in two patients (4%) in VX-659/TEZ/IVA arm (constipation in one patient and depression with suicidal ideation in another), whereas no SAEs occurred in the TEZ/IVA arm; no deaths were registered in either trial group.

A second phase 3, large, randomized, double-blind RCT (VX17-659-102) was conducted to assess the efficacy and safety of 24 weeks of VX-659/TEZ/IVA combination therapy in 385 subjects with CF older than 12 years and with F/MF genotype [76]. Bamocaftor combination therapy produced significant benefits in term of lung function also in F/MF subjects; it induced significant improvement in ppFEV1 of 14.0 and 14.2 percentage points at week 4 and at week 24, respectively. From the patient point of view, CFQ-R scores improved by 17.9 and 20.1 points at week 4 and week 24, respectively, for VX-659 based therapy [76]. In addition, at week 24, with Bamocaftor triple therapy, the number of pulmonary exacerbations and the sweat chloride concentration were significantly reduced versus placebo by 14% and −44.6 mmol/L, respectively. The nutritional status also improved significantly, with an increase in BMI of 1.11 with VX-659/TEZ/IVA over the placebo. Regarding safety, no difference was found in the percentage of patients with at least one AE between VX-659/TEZ/IVA combination therapy (90%) and placebo (93%) [76]. In the VX17-659-102 trial, AEs were almost mild to moderate and the most reported of them were upper respiratory tract infection, cough, sputum increase, nasopharyngitis, and headache in the Bamocaftor group and infective pulmonary exacerbation, cough, sputum increase, and headache in the placebo group [76]. The serious AEs were less frequent in the investigational group (6%) compared to placebo group (31%). There were no deaths in either trial group.

### 5.3. Proteostasis Pipeline

Proteostasis Therapeutics Inc. (PTI) developed a triple combination with a potentiator (PTI-808 or Dirocaftor), a corrector (PTI-801 or Posenacaftor), and an amplifier. The amplifier PTI-428 (or Nesolicaftor) demonstrated to increase the production of defective *CFTR* mRNA in vitro for many mutations, including F508del and some rare mutations without increased expression of the *CFTR* at the plasma membrane proving that *CFTR* remained dysfunctional [77,78]. A phase 1/2 clinical study (NCT03500263) in CF adults homozygous for F508del showed an improvement of 8% in ppFEV1 and a decrease of 29 mmol/L in sweat chloride after 4 weeks of treatment compared to the placebo dysfunctional [79].

## 6. Conclusions

*CFTR* modulators represent a turning point in the therapeutic approach to CF and have radically changed its nature by evolving from being an option for a niche of CF patients to representing a key therapeutic option beyond all expectations for the majority of CF patients, with increasingly promising results. Table 1 shows approved *CFTR* modulators with their indications, whereas Table 2 summarizes clinical trials on *CFTR*-modulators triple therapy.

Based on the known impact of the benchmark therapy Ivacaftor in a small subset of CF patients, the new combinations have been shown to change profoundly the clinical course of CF, leading to meaningful improvements in the lives of such a large proportion of people with CF heterozygous for F508del, especially if started in young children. Further studies are needed to extend the use of triple *CFTR* modulation therapy also for young children in order to prevent the irreversible effects of the disease and to provide patients with very rare mutations with a personalized approach to treatment. In addition, further studies could allow us to understand the efficacy and safety of new drugs in development (i.e., ABBV3067 and ABBV191 from Abbvie, ELX-02 from Eloxx Pharmaceuticals) as well as their synergy with the available molecules.

## Figures and Tables

**Table 1:** Approved *CFTR* modulators and their indications.

Modulators	Commercial Name	Approval Year	Responsive Mutations	Approved Ages
**Ivacaftor**	Kalydeco^®^ (EU/USA)	2012	G551D, S549N, G1244E, G178R, S1251N, G551S, G1349D, S1255P, R117H, E56K, K1060T, P67L, E193K, A1067T, R74W, L206W, G1069R, D110E, R347H, D579G, R1070Q, D1270N, D110H, R352Q, S945L, R1070W, R117C, A455E, S977F, F1074L, F1052V, D115H; 3849+10 kb C>T, 2789+5G>A, 3273-26A>G, 711+3A>G, E831X	≥4 months
**Lumacaftor-Ivacaftor**	Orkambi^®^ (EU/USA)	2015	Two copy of F508del	≥2 years
**Tezacaftor-Ivacaftor**	Symkevi^®^ (EU) Symdeko^®^ (USA)	2018	Two copy of F508del One copy of F508del in association with E56K, K1060T, P67L, E193K, A1067T, R74W, L206W, D110E, D110H, R347H, D579G, R1070Q, D1270N, R352Q, S945L, R1070W, R117C, A455E, S977F, F1074L, F1052V, D1152H, 3849+10 kb C>T, 2789+5G>A, 327326A>G, 711+3A>G	≥6 years
**Elexacaftor-Tezacaftor-Ivacaftor**	Kaftrio^®^ (EU) Trikafta^®^ (USA)	2020 (EU) 2019 (USA)	One copy of F508del	≥12 years

**Table 2:** Summary of clinical trials on *CFTR*-modulators triple therapy. Abbreviations: F/F = F508del/F508del genotype, F/MF = F508del/MF genotype.

Study					Patients Characteristics			Outcomes		
Study Identifier (NCT)	Name	First Author, Year	Phase	Investigational Drug	Age (Years)	Genotype	Number of Patients	ppFEV1 (%)	Sweat Chloride (mmol/L)	CFQ-R Score (Points)
NCT03227471	VX16-445-001	Keating, 2018	2	ELX/TEZ/IVA	≥18	F508del/F508del	28	+11 (*)	−39.6 (*)	+20.7 (*)
						F508del/MF	95	+13.8 (*)	−39.1 (*)	+25.7 (*)
NCT03224351	VX16-659-101	Davies, 2018	2	VX-659/TEZ/IVA	≥18	F508del/F508del	29	+9.7 (*)	−42.2 (*)	+19.5 (*)
						F508del/MF	88	+13.3 (*)	−51.4 (*)	+24.6 (*)
NCT03525548	VX17-445-103	Heijerman, 2019	3	ELX/TEZ/IVA	≥12	F508del/F508del	107	+10 (*)	−45.1 (*)	+17.4 (*)
NCT03525444	VX17-445-102	Middleton, 2019	3	ELX/TEZ/IVA	≥12	F508del/MF	403	+13.8 (*)	−41.8 (**)	+20.2 (**)
NCT03460990	VX17-659-103	-	3	VX-659/TEZ/IVA	≥12	F508del/F508del	111	+9.9 (*)	−48.7 (*)	+13.5 (*)
NCT03447249	VX17-659-102	-	3	VX-659/TEZ/IVA	≥12	F508del/MF	382	+14 (*)	−44.6 (**)	+20.1 (**)
NCT03691779	VX18-445-106	Zemanick, 2021	3	ELX/TEZ/IVA	6–11	F508del/F508del	29	+11.2 (**)	−70.4 (**)	+7 (**)
						F508del/MF	37	+9.1 (**)	−55.1 (**)	+6.9 (**)

(*): Outcome through week 4. (**): Outcome through week 24.

## Data Availability

Data sharing not applicable. The text has been written on findings reported in the mentioned References.
